# Emissivity evaluation of human enamel and dentin

**DOI:** 10.3389/fphys.2022.993674

**Published:** 2022-10-24

**Authors:** P. E. Lancaster, F. A. Carmichael, V. Clerehugh, D. S. Brettle

**Affiliations:** ^1^ Department of Restorative Dentistry, School of Dentistry, University of Leeds, Leeds, United Kingdom; ^2^ Department of Dental and Maxillofacial Radiology, Leeds Dental School, Leeds, United Kingdom; ^3^ Department of Medical Physics and Engineering, St. James’s University Hospital, Leeds, United Kingdom

**Keywords:** emissivity, thermal imaging, enamel, dentin, caries

## Abstract

**Background:** Human enamel and dentin temperatures have been assessed with non-contact infrared imaging devices for safety and diagnostic capacity and require an emissivity parameter to enable absolute temperature measurements. Emissivity is a ratio of thermal energy emitted from an object of interest, compared to a perfect emitter at a given temperature and wavelength, being dependent on tissue composition, structure, and surface texture. Evaluating the emissivity of human enamel and dentin is varied in the literature and warrants review. The primary aim of this study was to evaluate the emissivity of the external and internal surface of human enamel and dentin, free from acquired or developmental defects, against a known reference point. The secondary aim was to assess the emissivity value of natural caries in enamel and dentin.

**Method:** Fourteen whole human molar teeth were paired within a thermally stable chamber at 30°C. Two additional teeth (one sound and one with natural occlusal caries–ICDAS caries score 4 and radiographic score RB4) were sliced and prepared as 1-mm-thick slices and placed on a hot plate at 30°C within the chamber. A 3M Scotch Super 33 + Black Vinyl Electrical Tape was used for the known emissivity reference-point of 0.96. All samples were allowed to reach thermal equilibrium, and a FLIR SC305 infrared camera recorded the warming sequence. Emissivity values were calculated using the Tape reference point and thermal camera software.

**Results:** The external enamel surface mean emissivity value was 0.96 (SD 0.01, 95% CI 0.96–0.97), whereas the internal enamel surface value was 0.97 (SD 0.01, 95% CI 0.96–0.98). The internal crown-dentin mean emissivity value was 0.94 (SD 0.02, 95% CI 0.92–0.95), whereas the internal root-dentin value was 0.93 (SD 0.02, 95% CI 0.91–0.94) and the surface root-dentin had a value of 0.84 (SD 0.04, 95% CI 0.77–0.91). The mean emissivity value of the internal enamel surface with caries was 0.82 (SD 0.05, 95% CI 0.38–1.25), and the value of the internal crown-dentin with caries was 0.73 (SD 0.08, 95% CI 0.54–0.92).

**Conclusion:** The emissivity values of sound enamel, both internal and external, were similar and higher than those of all sound dentin types in this study. Sound dentin emissivity values diminished from the crown to the root and root surface. The lowest emissivity values were recorded in caries lesions of both tissues. This methodology can improve emissivity acquisition for comparison of absolute temperatures between studies which evaluate thermal safety concerns during dental procedures and may offer a caries diagnostic aid.

## 1 Introduction

### 1.1 Infrared imaging

Infrared imaging devices (such as thermal cameras) collect emitted infrared radiation and process this to provide a quantitative temperature measurement. The use of such an infrared imaging device, that collects, rather than projects, infrared radiation to provide an absolute temperature, is based on the theory that any tissue with a temperature above absolute zero emits infrared radiation. Absolute zero (0 K or −273.15°C) is where molecular motion is predicted to cease, resulting in no emittance of infrared radiation (Gaussorgues, 1994).

With technological developments, these devices are now more affordable and accessible, and, to provide accurate temperatures, certain parameters are required, which include the emittance-value or emissivity ( E ) of the object of interest.

### 1.2 Use of infrared imaging in dentistry

The use of non-contact infrared imaging devices to report temperature *via* thermography has benefits, which include data-collection from inaccessible areas, provision of areas of interest rather than a point of contact and avoidance of loss-of-contact during data-recording, and reduced cross-infection risk which, in turn, has a cost-benefit, compared to contact devices such as thermometers, thermocouples, and thermistors. The latter aspect of cross-infection is particularly beneficial for medical application.

With increased interest in the use of infrared imaging devices to assess the temperature of mineralized tooth tissue, it is relevant to assess the emissivity of each sample to report a valid temperature. Many *in vitro* studies have been carried out with thermal imaging devices to assess potential damage to both the soft and mineralized tooth tissue from temperature changes, e.g., during laser application (Launay, et al., 1987; Pogrel, et al., 1988; Anić, et al., 1993; Arima & Matsumoto, 1993; Neev, et al., 1993; Arrastia, et al., 1994; Anić and Matsumoto, 1995; Arrastia, et al., 1995; Machida, et al., 1995; Wilder-Smith, et al., 1995; Anić, et al., 1996a; Anić, et al., 1996b; Meyer & Foth, 1996; Neev, et al., 1996; Whitters & Strang, 2000; Yu, et al., 2000; Yamazaki, et al., 2001; Kishen, et al., 2003; Ishizaki, et al., 2004; Madura, et al., 2004; Wang, et al., 2005; Ana, et al., 2007; Da Costa Ribeiro, et al., 2007; Stock, et al., 2011; Da Silva Barbosa, et al., 2013; Uzunov, et al., 2014; Forjaz, et al., 2022), light-curing-composite (Al-Qudah, et al., 2005; Bouillaguet, et al., 2005; Aksakalli, et al., 2014; Jo, et al., 2019; Mouhat, et al., 2021), endodontic treatment (McCullagh, et al., 1997; McCullagh, et al., 2000; Behnia & McDonald, 2001; Lipski and Zapałowicz, 2002; Lipski & Woźniak, 2003; Lipski, 2004; Lipski, 2005a; Lipski, 2005b; Lipski, 2006; Hsieh, et al., 2007; Ulusoy, et al., 2015; Diegritz et al., 2020; Podolak, et al., 2020), pin-placement (Biagioni, et al., 1996), post-removal (Budd, et al., 2005; Lipski et al., 2010a), cavity preparation and restoration (Carson, et al., 1979; Lipski et al., 2020), caries assessment (Liu, et al., 2021; Roointan, et al., 2021), and bleaching (Gontijo, et al., 2008; Kabbach, et al., 2008).

**FIGURE 1:**
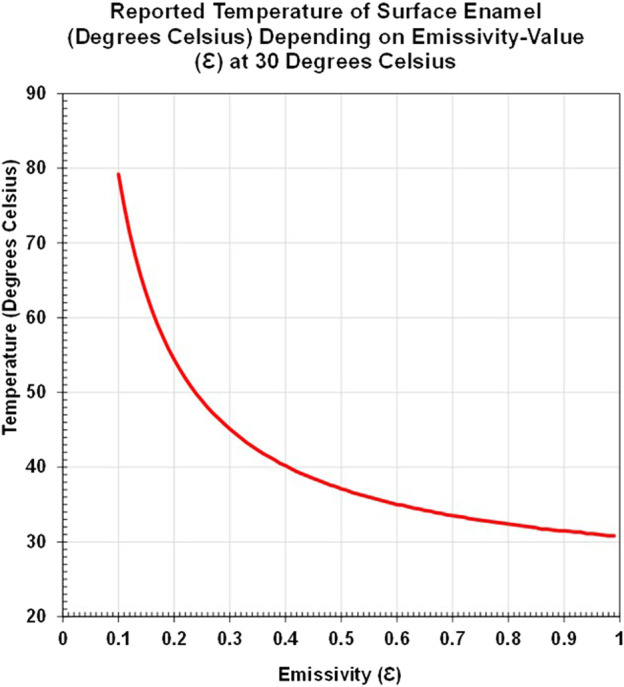
Line graph of changing temperature with emissivity of human enamel at 30°C.

A smaller number of *in vivo* studies have also assessed the temperature of tooth tissue and oral soft tissue, e.g., investigation of infection (Crandell & Hill, 1966; Pedreira, et al., 2016; Aboushady, et al., 2021), vitality (Hartley, et al., 1967; Pogrel, et al., 1989; Kells, et al., 2000b; Mendes, et al., 2020), composite curing (Hussey, et al., 1995), and tooth temperature after laser application (Arrastia, et al., 1994). A small infra-camera has been developed and used clinically to assess root caries *in vivo* (Yang et al., 2020), progressing the application of infrared imaging in clinical dentistry. However, recognition of the emissivity value of mineralized tooth tissue and the method of calculation is very varied within the dental literature, with values ranging from 0.65 to 1.0 (Table 1). Some studies report the method of emissivity assessment, some reference other studies for the transferred value, some have previously reported a value in a study, and others may not report a methodology due to space available in the article or due to prior unpublished work ascertaining the emissivity value. Diegritz, et al., 2020, stated ‘*it is of vital importance to determine the emissivity of the object of interest as it will affect the radiation emitted and, therefore, also affect the temperature measurement*’. Without the emissivity value, the temperature reported may be of limited value, as seen from the range of temperatures in Figure 1.

**TABLE 1:** Emissivity values sourced in literature to July 2022 for mineralized human tooth tissue.

Emissivity (ε)	Author	Year	Tissue (E, D, R)	*In vivo* (VV) *In vitro* (VT)	Sample S or W	Calculation method
1.0	Jo, et al.	2019	D	VT	W	Default setting of the program
0.98	Mouhat, et al.	2021	D	VT	W	Previous study by same group 2017 with no method given
0.98	Kaneko, et al.	1999	E demineralized	VT	W	Assumed
0.98	Preoteasa, et al.	2010	E	VV	W	Referenced Voicu et al., 2009
0.97	Soori, et al.	2020	E	VT	W	Thermocouple comparison 40°C–60°C
0.97	Meyer & Foth	1996	E & D	VT	S	Assumed
0.96	Soori, et al.	2020	E	VT	W	Thermocouple comparison 20°C–40°C
0.96	Lancaster, et al.	2017	E & D	VT	S	.
0.92	Liu, et al.	2021	E	VT	W	Comparison with the original reference image to ambient temperature
0.92	Soori, et al.	2020	D	VT	W	Thermocouple comparison 40°C–60°C
0.92	Dabrowski, et al.	2000	E	VT	W	Reflection method
0.92	Lee, et al.	2016a	R	VT	W	.
0.92	Lin, et al.	2010a	D	VT	S	Manufacturer’s guide
0.91	Forjaz, et al.	2022	R	VT	W	Considering emissivity to be equal to 0.91
0.91	Soori, et al.	2020	E	VT	W	Thermocouple comparison 20°C–40°C
0.91	Mendes, et al.	2020	E	VV	W	Resultant from the vestibular surface of the assessed teeth
0.91	Podolak, et al.	2020	R	VT	W	Referenced Kells et al. (2000a)
0.91	Arslan, et al.	2018	R	VT	W	Calibrated to the specific root
0.91	Lee, et al.	2016b	E	VT	S	.
0.91	Lipski, et al.	2020	Roof of pulp chamber	VT	W	Referenced Kells et al. (2000a)
0.91	Lipski	2005a	R	VT	W	Referenced Kells et al. (2000a)
	Lipski, et al.	2010a				
	Lipski, et al.	2010b				
0.91	Lipski	2005b	R	VT	W	·
0.91	Lipski	2006	R	VT	W	Camera calibration
0.91	Ana, et al.	2007	E & D	VT	S	·
0.91	Da Costa Ribeiro, et al.	2007	R	VT	W	Referenced McCullagh et al. (2000)
0.91	Da Silva Barbosa, et al.	2013	E Deciduous	VT	S	Referenced Ana et al. (2007)
0.91	Kabbach, et al.	2008	RD	VT	W	·
0.91	Lin, et al.	2010a	E	VT	S	Manufacturer’s guide
0.91	Kilic, et al.	2013	R	VT	W	Referenced Lipski et al. (2010a) & Kells et al. (2000a)
0.91	Ulusoy, et al.	2015	R	VT	W	·
0.9	Diegritz, et al.	2020	R	VT	W	Thermal comparison with thermocouple
0.84	Paredes, et al.	2018	E	VT	W	Reference tape
0.8	Neev, et al.	1993	D	VT	S	Black paint assumed-emissivity 1
0.65	Kells, et al.	2000a and b	E	VT/VV	W	Spot-measurement from within the hot oven and software
0.65	Kells, et al.	2000a and b	E	VT/VV	W	Spot-measurement from within the hot oven and software

E, enamel; D, dentin; R, root; RD, root-dentin; VV, *in vivo*; VT, *in vitro*; S, sectioned flat surface of a slice; W, whole tooth.

### 1.3 Emissivity

When the thermal stability of an object is achieved, there is a balance between the radiation entering and emitted from an object. To maintain this thermal equilibrium, when the energy is absorbed, energy must also be released, which is the emitted radiation ( E) . This amount is dependent on the temperature, wavelength, material composition, and surface texture, as well as the viewing angle (Gaussorgues, 1994; Vollmer & Möllmann, 2010), although the visible color of the object is not influential (Hardy, 1934).

The maximum potential value for the emitted radiation is 1 and is described for an idealistic blackbody, which theoretically absorbs all incident radiation and can subsequently emit all of it. Commercially available blackbodies have reported emittance values of 0.98 or 0.99 rather than 1, which is the highest empirical value achievable (Vollmer & Möllmann, 2010). The emissivity value, which is unitless and can range from 0 to 1, is the ratio of the emitted radiation from the surface-of-interest, compared to that emitted by a blackbody at the same wavelength and temperature.

Emissivity calculation methods may involve a blackbody, but this is expensive and often unavailable. Temperature measurement has also been described (Webb, 1991) but requires bespoke software to compute the value and an environment with known stable temperatures to assess the samples, whereas a known reference allows emissivity calculation using the thermal camera’s software and is accessible to all with such a camera. Stability of the thermal environment is still an important consideration. An affordable and accessible reference, which may be a black paint or black tape with reported emissivity, e.g., 3M Scotch Super 33 + Black Vinyl Electrical Tape (≈$12 for 20 m) has a recorded value of 0.96 (British Standards, 2008; FLIR ThermaCAM™ Researcher Professional, 2010).

Emissivity may vary due to composition, tissue structure, surface contour (as seen with occlusal fissures and the natural curve of a whole tooth crown, compared to the flat internal surface of a tooth slice), and the tissue temperature and wavelength-collection of different thermal devices. Transfer of emissivity values, as seen in multiple articles (Lipski, 2005a; Da Costa Ribeiro, et al., 2007; Lipski, et al., 2010a; Lipski, et al., 2010b; Preoteasa, et al., 2010; Da Silva Barbosa, et al., 2013; Kilic, et al., 2013; Lipski et al., 2020; Podolak, et al., 2020), requires care, and a standardized approach to assess the emissivity of tooth tissue would be beneficial. As seen in Figure 1, a difference in emissivity can lead to large temperature differences. In a stable environment of ≈30°C, the surface enamel may be reported to have a temperature of 79.2°C, with an emissivity value of 0.1, whereas a temperature of 30.8°C is reported with an emissivity value of 0.99, a difference of 48.4°C. If used for assessing temperature differences with new equipment or comparing with threshold values for safety, this can lead to misleading outcomes if incorrectly applied.

Two studies were sourced in a literature search of Medline, Web of Science, and Scopus databases up to July 2022, which focused solely on emissivity values of human mineralized tooth tissue (Dabrowski, et al., 2000; Soori, et al., 2020). The recent study by Soori, et al. (2020) reported experimental estimation of human enamel and dentin emissivity to be 0.96 ± 0.01 and 0.92 ± 0.01, respectively, between 20 and 40°C, 0.97 ± 0.01, and 0.93 ± 0.01, respectively, between 40 and 60°C, which lies within previously reported values from thermal studies (Table 1) and reports the variances are due to compositional and structural differences in the tissues, as well as surface quality. Thermocouples were used internally on the teeth, which could not be applied clinically. Dabrowski, et al. (2000) reported an enamel emissivity of 0.92 and also recognized the kind of material that affects this parameter. To assist the clinical evaluation of emissivity, a non-invasive method is desirable.

### 1.4 Composition, structure, and surface texture of human teeth–health and caries

The composition, structure, and surface texture of the human tooth can be variable, and this will be reviewed considering the implications for the range of emissivity values for human enamel, both external surface-enamel and internal enamel visible when a tooth is sliced, internal crown and root-dentin, as well as external surface root-dentin. The impact of dental caries on the composition, structure, and surface texture is also considered as emissivity may offer an additional diagnostic tool for detecting demineralized tissue.

The human tooth has a clinical crown visible in the oral cavity and a root, invisible in the oral cavity, which attaches to the alveolar bone of the jaw *via* a soft tissue, the periodontal ligament. There are three mineralized tissues that compose a tooth: enamel, dentin, and cementum, and one soft tissue internally encased in the mineralized tooth tissue: the pulp.

Developmentally, enamel has an epithelial origin, whereas dentin, cementum, and the alveolar bone are mesenchymal in origin. Despite different origins and final structure, which relates to tissue function, the underlying mineral of the tooth enamel, dentin, and cementum is quite similar, being calcium hydroxyapatite (Ca_10_(PO_4_)_6_(OH)_2_).

#### 1.4.1 Enamel

During tooth development, ameloblasts produce enamel (amelogenesis) after dentin formation (dentinogenesis), commencing from odontoblasts within the central pulp-tissue. The enamel forms the external surface of the tooth exposed in the oral cavity and has a varying thickness depending on age, sex, genetics, developmental anomalies, location, and physiological and pathological wear.

The enamel is a very hard and organized tissue primarily composed of inorganic calcium hydroxyapatite (Ca_10_(PO_4_)_6_(OH)_2_) (96% by weight when mature— Goldberg, et al., 2011) and soft organic tissue, such as the non-collagenous structural proteins, amelogenin, ameloblastin, and enamelin, as well as proteinases, kallikrein-related peptidase-4 (KLK4) and matrix metalloproteinases-20 (MMP20) (Simmer, et al., 2012; Bartlett, 2013; Smith, et al., 2017). As the enamel matures, mineral content increases with the loss of protein and water, most likely resulting in a non-homogenous mineral distribution (Cuy, et al., 2002; He, et al., 2010; Zheng, et al., 2013), although Glas (1962) reported little mineral change across the enamel. This non-homogenous distribution can lead to different properties of the tissue in different places.

The structure of the enamel and dentin is quite different and can explain their different properties.

The initial layer of enamel interdigitates with dentin and has no rods. Rods are produced later with thousands of crystallites which vary in size, increasing with maturity (≈5 µm cross-section diameter—Meckel, et al., 1965), up to 40,000 crystallites per bundle, varying in thickness from 30 nm × 30 nm (Robinson, et al., 2003) and width 26 nm × 68 nm (Kerebel, et al., 1979), producing smooth surfaces extending the full width of enamel to the surface (Daculsi & Kerebel, 1978; Daculsi, et al., 1984). Between the rods are an organic rod-sheath and an interrod area of differently orientated crystals. Human rod cross-sections are key-hole-shaped, with a circular head and elongated tail. Adjacent enamel crystals have been reported to be misoriented with a range of 0–30^o^ and a mean of 2–8^o^ (Stifler et al., 2021), and this relates with the reported hardness of this unique tissue, which is the hardest tissue in man. This change in orientation produces Hunter–Schreger bands which are observed to be horizontal at the side of a cusp, as the rods may be viewed in cross-section or longitudinal. At the occlusal surface, a final aprismatic enamel layer (30–70 µm) overlies the radial enamel, which has rods parallel to each other and perpendicularly orientated to the surface (Speirs, 1971; Whittaker, 1982; Maas & Dumont, 1999; Popowics, et al., 2004; Cui & Ge, 2007; Risnes & Li, 2018), unlike the body of enamel. The post-secretory maturing ameloblasts secrete proteases which degrade the matrix, reducing the extra-cellular content from 20 to 0.4–0.6%, and with crystal growth, the peptides are removed (Goldberg, et al., 2014).

As seen, there is a difference between the cross-section internal structure of enamel compared with the outer aprismatic layer, followed by the outer radial prismatic layer and finally the inner enamel with Hunter–Schreger bands, compared to the sound intact outer surface enamel, which may be totally aprismatic (40%–47%) (Risnes & Li, 2018), with areas of the ends of radial prisms visible, or, subject to greater enamel loss, may show areas of Hunter–Schreger bands from the inner enamel (Whittaker, 1982). With sufficient enamel loss, the inner layer may be seen in the transverse section clinically. The prism-free enamel is less rough than that with prisms, especially when etched. The surface enamel on unerupted teeth shows primarily small crystals (5 nm), which are loosely packed, with a few larger plate-like crystals (1.0µm—50 nm). Following the eruption, such crystals were not observed, and aprismatic enamel had crystals of 40 nm or more (Palamara et al., 1980). The surface enamel may be fissured occlusally or curved over the cusps and buccal and lingual surface, whereas the inner surface will be flat following slicing.

Enamel is deposited rhythmically by each ameloblast producing individual prisms, and collectively the striae of Retzius demonstrate the overall enamel deposition and growth, which results in the presence of enamel surface ridges—the perikymata. This is the interface with the oral cavity and will be examined clinically and thermographically for the external surface of the enamel. All of these enamel types are in a different orientation internally compared to externally, with or without wear, and this may affect the emissivity value of the tissue due to surface texture.

#### 1.4.2 Dentin

Beneath the enamel lies the dentin, which forms the bulk of the tooth’s mineralized tissue, both in the crown and the root, which is less mineralized than enamel at 70% by weight (Goldberg, et al., 2011). Physiological deposition of dentin continues throughout the vital life of the tooth from odontoblasts at the outer edge of the pulp, which moves toward the center of the tooth, secreting the collagenous predentin matrix 15–20 µm thick and commencing mineralization of intra- and extra-fibrillar crystals in a ratio of 25–30% and 70–75%, respectively (Kinney, et al., 2001; Kinney, et al., 2003; Balooch, et al., 2008), which is dissimilar to enamel but similar to bone (Bonar, et al., 1985; Pidaparti, et al., 1996). Development of matrix vesicles from odontoblasts is reported early in dentin-formation, which is similar to bone and may contribute to mineralization of dentin (Goldberg, et al., 2011).

There are multiple types of dentin, each with structural and compositional differences. Mantle dentin is the first formed coronally, without tubules as the odontoblast process develops later. In the root, a Tomes granular layer with interglobular spaces, with or without the hyaline Hopewell–Smith layer, is deposited, and tubules are rare in any peripheral dentin (Goldberg, et al., 2011). Primary dentin (circumpulpal dentin) is composed of three dentin types: intertubular dentin may account for up to 90% with 30% mineral and Type I collagen as the main protein, intratubular dentin and peritubular dentin in the human may account for 10%–20%, with 95% mineral with no collagen, and the latter two are often considered one tissue. This is variable depending on the location. The crystals of intertubular dentin form from two plates and may be 2–5 nm thick and 60 nm long, whereas peritubular dentin crystals of 25 nm appear isodiametric, and, when viewed with higher resolution dimensions are 36 nm × 25 nm × 9.75 nm (Goldberg, et al., 2011). Once erupted and contacting the opposing dentition, physiological deposition of secondary dentin continues at a slower rate than that of primary dentin.

Tertiary dentin may be produced in response to a pathological stimulus, e.g., caries, which offers additional protection of the pulp from the original odontoblast (reactionary dentin) or from odontoblast-like cells differentiated from pulpal stem cells following the death of the original odontoblast. This dentin differs from physiological and reactionary dentin as the odontoblast-like cells do not have an odontoblast process around which a tubule forms (Smith, et al., 1995). The odontoblast process may extend up to 1 mm into the dentin, resulting in cellularity, unlike enamel (Pashley, 1996). The tubule diameter varies across the dentin-thickness, being larger at the pulp-face (2.5–3.5 µm) than at the periphery of the amelodentinal junction (0.6–1.5 µm) (Fearnhead, 1957; Linde and Goldberg, 1993; Pashley, 1996; Montoya, et al., 2015).

#### 1.4.3 Root

The root of the tooth is not normally visible intra-orally when held in a healthy periodontium, and the outer surface is covered with a thin layer of the mineralized tissue cementum. There are several types of cementum—acellular, cellular, mixed, and acellular afibrillar—composed of small mineralized plates similar to bone (Yamamoto et al., 2010; Nanci, 2012).

### 1.5 Properties

These compositional and structural differences between enamel and dentin can impact their properties, e.g., conductivity, diffusivity, and emissivity.

Thermal conductivity of the enamel is reported to range between 0.65 and 0.93 W m ∙ K (Lisanti and Zander, 1950; Phillips et al., 1956; Craig and Peyton, 1961; Braden, 1964; Lin, et al., 2010b; Lancaster, et al., 2017), indicating enamel is an insulator offering thermal protection to the underlying dentin and pulp. Consideration was given to the orientation of enamel rods (parallel to or perpendicular to) in one study (Soyenkoff and Okun, 1958), but it did not impact the outcome, as both orientations produced a value of 0.65 W m ∙ K. A later study did report a higher value (0.93 W m ∙ K) when analyzing parallel rods. However, neither study had large sample sizes (2 v 7 respectively), and each used different temperatures, i.e., 26–29°C v 50°C, to record thermal conductivity with different devices, e.g., thermistor v thermocouple.

The thermal conductivity of dentin is reported to range between 0.108 and 0.959 W m ∙ K (Lisanti & Zander, 1950; Phillips, et al., 1956; Soyenkoff & Okun, 1958; Craig & Peyton, 1961; Braden, 1964; Brown et al., 1970; Fanibunda and de Sa, 1975; Minesaki et al., 1983; Fukase et al., 1992; Panas et al., 2003; Little, et al., 2005; De Magalhaes et al., 2008; Lin et al., 2010b; Lancaster, et al., 2017), which is a 10-fold change rather than a 1.5-fold change for enamel. Dentin structure may have greater variability than enamel, especially when reviewing crown- and root-dentin and young and mature dentin. Tubule orientation was viewed in parallel and perpendicular, giving similar results with a range of 0.4–0.6 W m ∙ K.

Human enamel thermal diffusivity ranges from 0.23 to 0.47 × 10^−6 ^m^2^/s, approaching twice the value of human dentin at 0.18–0.26 × 10^−6 ^m^2^/s (Lisanti & Zander, 1950; Phillips et al., 1956; Soyenkoff & Okun, 1958; Craig & Peyton, 1961; Braden, 1964; Brown et al., 1970; Fanibunda and de Sa, 1975; Minesaki et al., 1983; Fukase et al., 1992; Panas et al., 2003; Little, et al., 2005; De Magalhaes et al., 2008; Lin, et al., 2010b; Lancaster, et al., 2017).

### 1.6 Caries

During the life of a human tooth, there is likely to be a physiological and pathological impact on the tissue from abrasion and/or attrition, which may smooth the surface or expose the underlying structure clinically; demineralization and remineralization due to erosion or caries with an ionic exchange, e.g., calcium, phosphate, magnesium, and fluoride can result in a changeable composition (Fejerskov, 1997).

Immature teeth may be susceptible to caries due to increased porosity, incomplete mineralization, and plaque accumulation (Carvalho, 2014). Caries initially has a subsurface effect with an intact surface layer of 20–50 µm, which may have surface roughness and pore volume of 1% (Darling, 1958) and an increasing porosity below the body of the lesion (5%–25%), with increased loss of mineral, e.g., magnesium, which may arrest or progress, leading to breakdown of the surface and underlying tooth structure. The crystal size is affected, reducing to 10–30 nm in the body of the lesion, and also alters the structure and composition of the enamel, which may also affect the emissivity value.

Dentin may also be affected if caries progresses, leading to bacterial invasion and a zone of destruction and degradation of the organic matrix with loss of extrafibrillar mineral initially (Frank, 1990; Pugach, et al., 2009).

Coronal caries can be visually and radiographically characterized for research using the International Caries Detection and Assessment System (ICDAS) and ICDASII, with scores for enamel and dentin ascertained by assessing the surface changes of a tooth, which has been related to the histological extent of the lesion (Pitts et al., 2013). This improves the consistency of reporting caries compared to a basic indication of the presence or absence of decay. For the unrestored tooth, caries can be visually scored from 0 to 6, ± for activity status, as well as by merged variations to simplify the classification process–Tables 2, 3.

**TABLE 2:** ICDAS II Merged Caries Codes adapted from Pitts et al. (2013).

ICDAS II Caries code	Merged codes	Description
0+/−	0−no caries evident	Sound tooth—no or questionable changes in enamel translucency viewed clean and after prolonged air-drying of 5 s
1+/−	A+/−initial stage decay	Initial stage of caries—first visible change in the enamel observed as a caries opacity or discoloration of pits after drying with air, not consistent with sound enamel. No surface breakdown or dentin shadowing.
2+/−		Change in enamel visible on moist enamel, extending beyond pits
3+/−	B+/−moderate decay	White or brown spot lesion with localized enamel destruction, without visible dentin
4+/−		Underlying dentin shadow with or without localized enamel destruction
5+/−	C+/−extensive decay	Clear cavity, less than half of the dental surface, in enamel showing dentin
6+/−		Extensive cavity, more than half the tooth surface, which is deep and wide, extending into the dentin

**TABLE 3:** ICDAS Radiographic Scores adapted from Pitts et al. (2013).

ICDAS Radiographic score	Codes	Description
0	No radiolucency	
RA: initial stages	RA 1	Radiolucency outer half enamel
	RA 2	Radiolucency inner half of enamel ± enamel dentin junction
	RA 3	Radiolucency limited to outer third of dentin
RB: Moderate stages	RB 4	Radiolucency reaching middle third dentin
RC: Extensive stages	RC 5	Radiolucency reaching inner third dentin and clinically cavitated
	RC 6	Radiolucency into pulp and clinically cavitated

The impact of natural caries on human enamel is reported to reduce the thermal conductivity for both enamel (0.22 W m ∙ K) and dentin (0.24 W m ∙ K) (Lancaster, et al., 2017), and this may also affect the emissivity value.

### 1.7 Aims

The primary aim of this study is to evaluate the emissivity of the external and internal surface of human enamel and dentin from a known reference of 3M Scotch Super 33 + Black Vinyl Electrical Tape. The secondary aim is to assess the emissivity of natural caries in internal enamel and dentin.

The hypothesis for this study is that emissivity will be the same for all enamel and dentin types, whether sound or carious.

## 2 Materials and methods

Ethical approval was gained from Leeds Dental Institute Research Tissue-Bank for all teeth. Two teeth, one sound and one with a natural carious lesion, were radiographed (70kV/7mA/0.16s) with a Focus 50420 radiographic unit (Instrumentarium Dental TUUSULA, Finland) sliced bucco-lingually at 1-mm intervals with an Accutom-5 (Struers, Copenhagen, Denmark) and polished with an 800-grit abrasive sheet, while being cleansed with distilled water as necessary. The slices were immersed in distilled water and stored flat in boxes, which were refrigerated until needed.

**FIGURE 2:**
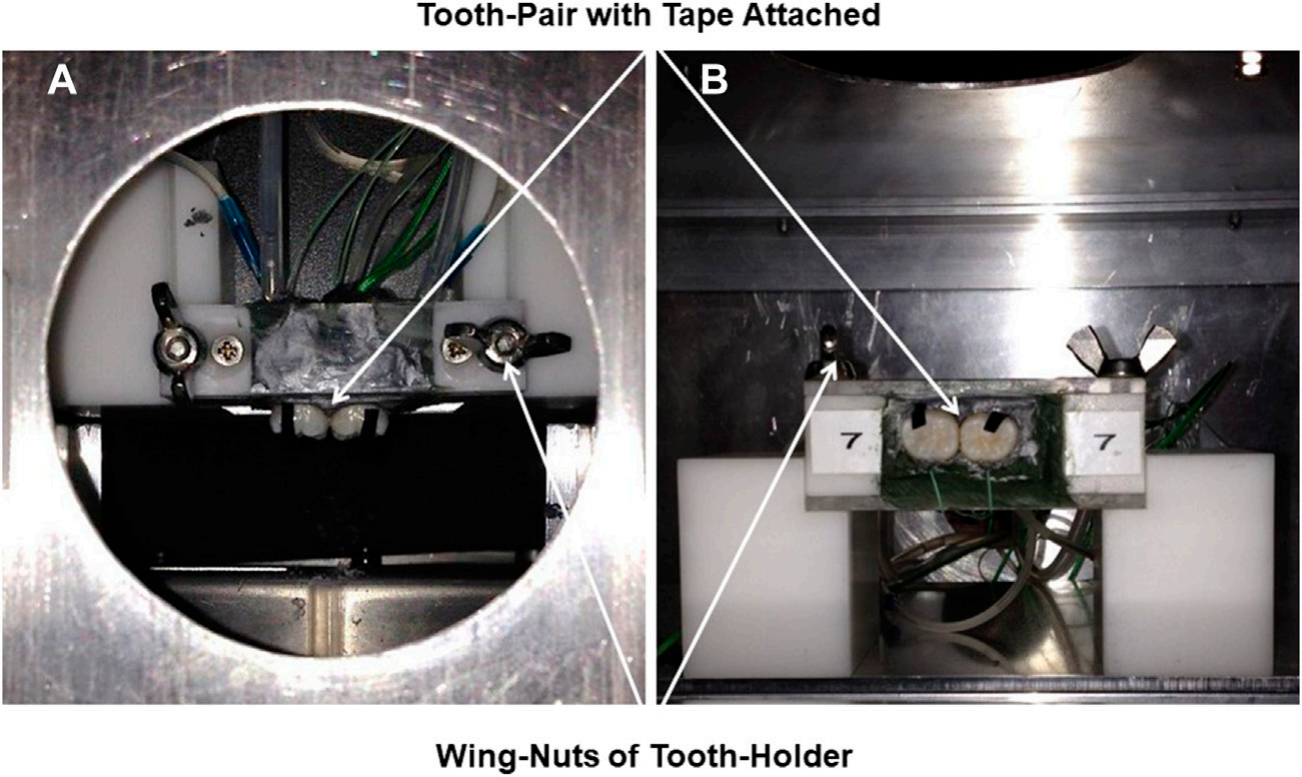
**(A)**: Vertical and **(B)**: horizontal close-up of the tooth-pair unit secured by wing-nuts to the rigid-stand in the aluminium cube. 3M Scotch Super 33+ Black Vinyl Electrical Tape attached to tooth-surface for emissivity-reference-point of 0.96.

Fourteen whole human molar teeth were held as pairs within a purpose-built unit, numbered 1 to 7, for ease of placement and removal from a fixed, rigid aluminum frame attached to an aluminum cube at a focal distance of 8 cm from the thermal camera. The frame had two supports to hold the paired units of teeth with the Tape attached, which were secured with wing-nuts (Figures 2A,B). The pairs of teeth were embedded within a simulated alveolar bone of Aluwax (www.Aluwaxdental.com) and secured with Green Stick Compound (www.kerrdental.com).

A thermal camera (FLIR SC305 with x4 lens, 100 µm spatial resolution) was attached to the aluminum cube with macro- and micro-thermal-regulation (previously described by Lancaster, et al., 2017), with a focal distance of 8 cm to the object of interest. Parameters of reflected apparent temperature (assessed with a thermal image of crumpled aluminum foil) were 27.2°C with a humidity of 50% (Prime Capsule Data Logger—www.perfect-prime.com) at a stable cube temperature of 22°C for the slices and 34.6°C with a humidity of 29% at a stable cube temperature of 30°C for whole teeth.

**FIGURE 3:**
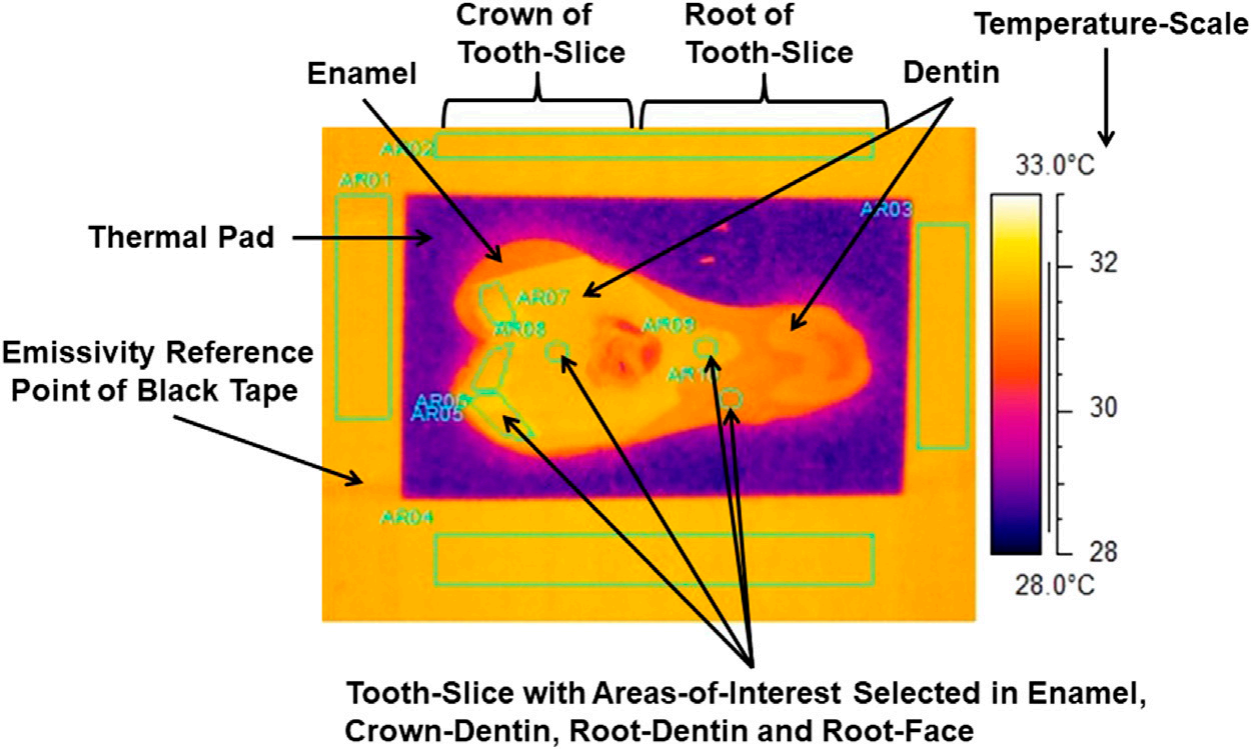
Thermograph with areas-of-interest selected in internal enamel, crown-dentin, root-dentin and root-face of a slice of tooth. Emissivity-reference-point provided by 3M Scotch Super 33+ Black Vinyl Electrical Tape.

A Bibby Hotplate Techne DB-2TC (www.bibby-scientific.com) with an aluminum block was secured within the cube and provided a stable heat source of 30°C. A hand carrier with a copper baseplate (0.5 mm × 50 mm × 50 mm) and attached thermal tape (6 W m∙K; www.thegamebooth.co.uk) transported the paper-dried tooth slices for heating on the heated aluminum block (Figure 3). The 3M Scotch Super 33 + Black Vinyl Electrical Tape was used for the known emissivity reference-point of 0.96 (ε = 0.96). The sliced samples were heated within the cube for 20 min, when thermal equilibrium was achieved, and emissivity was calculated against the reference Tape using the thermal camera software (ThermaCAM Researcher Professional 2.10).

A stable thermal environment of 30°C was provided within the cube for the whole teeth, and, when in thermal equilibrium, the enamel-surface emissivity was assessed from the thermographs against the reference Tape attached to each tooth.

Data were processed and analyzed with ThermaCAM Researcher Professional 2.10 Software, which was also embedded in a Macro-enabled Microsoft Excel File (Microsoft^®^), and 95% confidence interval and Intraclass correlation coefficient were calculated in IBM SPSS Statistics Version 23.

## 3 Results

### 3.1 Descriptive data of teeth

The fourteen whole molar teeth evaluated for the emissivity of surface enamel comprised three upper-third molars, four lower-third molars, one lower-second molar, five upper-first molars, and one lower-first molar. Nine were donated by females and five by males. The mean age of donors was 16 years 11 months (range 10–28 years). Twelve donors were of white ethnic origin, and two were unknown.

A lower-third molar from a female donor, aged 18 years, of unknown ethnic origin, and an upper-third molar, also from a female donor, aged 28 years, also of unknown ethnic origin, provided five slices from each tooth. The caries lesion of the whole upper third molar tooth was classified as ICDAS II caries score 4 and radiographic score RB4.

### 3.2 Thermal equilibrium

**FIGURE 4:**
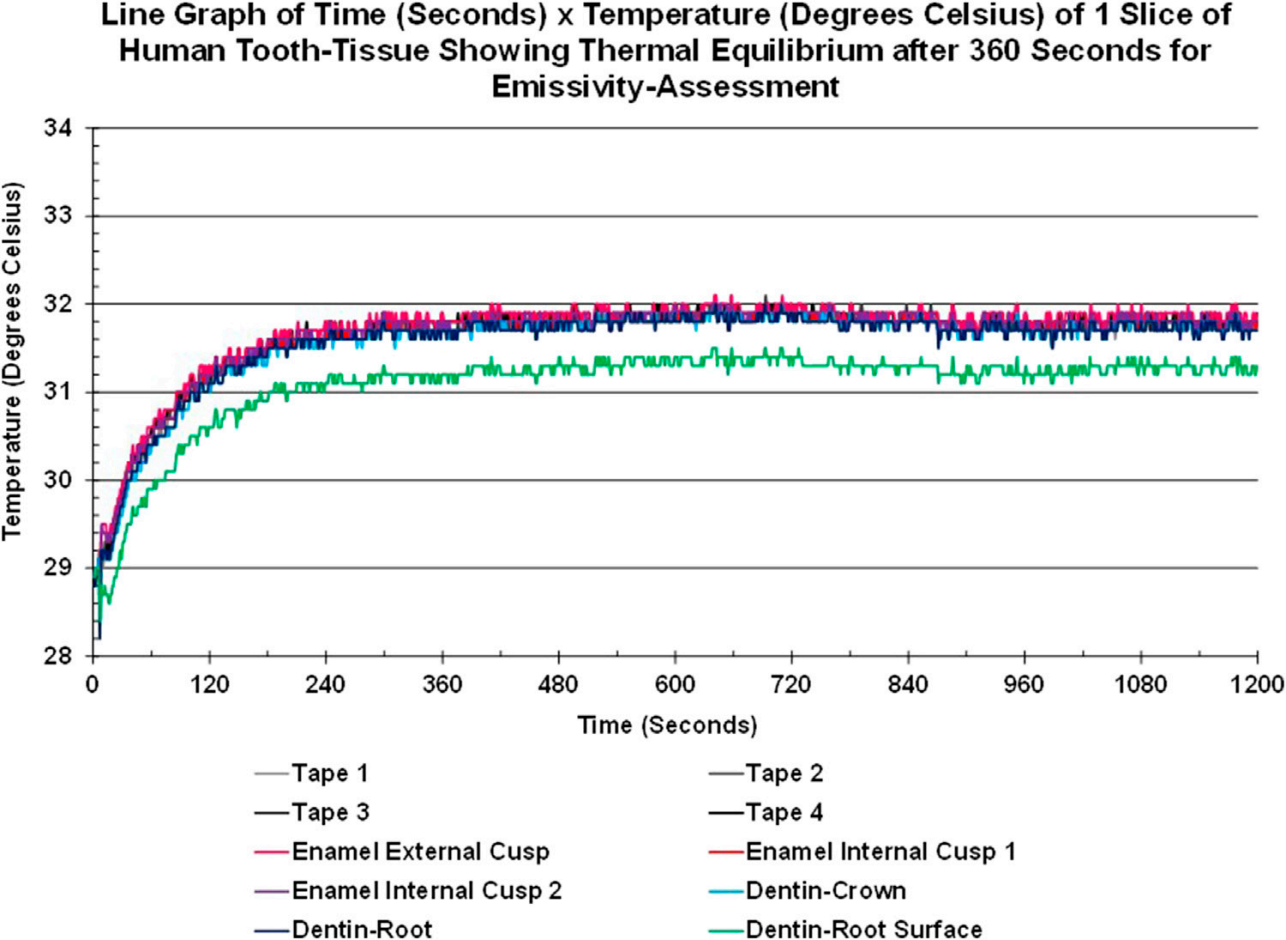
Line graph showing the Time-Temperature Curves of areas-of-interest of enamel, crown-dentin, root-dentin, root-surface-dentin of a slice of human tooth-tissue and tape areas-of-interest achieving thermal equilibrium within the cube after approximately 6 min and remaining stable over the next 14 min.

Thermal equilibrium was achieved for each sample prior to emissivity assessment. As seen in Figure 4, the tooth slice warmed from the base temperature (lowest value of 28.2°C for the root surface) to 31.8°C for all tissues and materials bar one in approximately 360 s (6 min) and remained stable (±0.3°C) for the rest of the 20-min sequence, at the end of which the emissivity value was calculated. The lowest value found was for the root surface of dentin (green line), which stabilized between 31.5°C and 31.1°C.

### 3.3 Enamel and dentin emissivity

Table 4 and Table 5 show the results for the emissivity value by sample location (external or internal) and sub-sample slice with and without caries and descriptive statistics for emissivity values of sound external enamel, internal enamel, dentin, root-face dentin, and caries-affected internal enamel and dentin, respectively.

**TABLE 4:** Emissivity value by sample location (external or internal) and sub-sample slice with and without caries.

Whole samples (*n* = 14)	External enamel	Sub-sample slices (*n* = 2)	Internal enamel	Dentin			Internal enamel caries	Internal dentin caries
				Crown	Root	Root-face		
1	0.955	15a	0.973	0.950	0.921			
2	0.965	15b	0.971	0.917	0.926			
3	0.970	15c	0.957	0.943	0.940	0.838		
4	0.970	15d	0.991	0.960	0.943	0.902		
5	0.959	15e	0.951			0.827		
6	0.966	16a	-			0.796		
7	0.961	16b	0.960	0.897	0.907			0.714
8	0.967	16c	0.978	0.930	0.910		0.781	0.658
9	0.963	16d	0.976	0.931	0.926		0.849	0.809
10	0.959	16e	0.992	0.960	0.960			
11	0.958							
12	0.954							
13	0.940							
14	0.948							

**TABLE 5:** Descriptive statistics for emissivity values of sound external enamel, internal enamel, dentin, root-face dentin, and caries-affected internal enamel and dentin.

Tissue	*n* =	Mean	Std. deviation	Std. error mean	95% Confidence interval
External enamel	14	0.96	0.01	0.002	0.96–0.97
Internal enamel	2 (nine slices)	0.97	0.01	0.005	0.96–0.98
Internal dentin					
Crown	2 (eight slices)	0.94	0.02	0.008	0.92–0.95
Root	2 (eight slices)	0.93	0.02	0.006	0.91–0.94
Root face	2 (four slices)	0.84	0.04	0.022	0.77–0.91
Internal enamel caries	1 (two slices)	0.82	0.05	0.034	0.38–1.25
Internal dentin caries	1 (three slices)	0.73	0.08	0.044	0.54–0.92

Comparison of enamel emissivity produced similar outcomes for the external enamel (x̄ = 0.96, SD 0.01) and internal enamel (x̄ = 0.97, SD 0.01), both falling within the 95% confidence interval. Repeat sequences of two slices gave an Intraclass Correlation Coefficient of 0.86 for the internal enamel.

Comparison of internal caries enamel emissivity (x̄ = 0.82, SD 0.05) indicated a potential difference in emissivity, falling below the 95% confidence interval of 0.96–0.98 for sound internal enamel.

Comparison of the internal crown (x̄ = 0.94, SD 0.02) and root dentin (x̄ = 0.93, SD 0.02) emissivity produced similar outcomes, both falling within the 95% confidence interval. Repeat sequences of two slices gave an ICC of 0.86 for internal root dentin.

Comparison of the external root-face dentin (x̄ = 0.84, SD 0.04) with the internal crown and root dentin indicates potential differences in emissivity, with the root-face falling below the confidence interval of both internal crown and root dentin.

Comparison of internal carious crown dentin (x̄ = 0.73, SD 0.08) indicated potential difference with the respective sound tissue, falling below the 95% confidence interval of 0.92–0.95 and having the lowest of all emissivity values.

Based on the above-mentioned findings, the study hypothesis was rejected.

## 4 Discussion

The British Standards and Manufacturer’s Infrared Thermography Handbook (British Standards, 2008; FLIR ThermaCAM™ Researcher Professional, 2010) provide a simple and recognized method of calculating emissivity by using a reference point of known emissivity, e.g., 3M Scotch Super 33 + Black Vinyl Electrical Tape.

Multiple samples (14) were used in this study to assess the emissivity value of the external surface of the enamel of whole teeth compared to one sample from Kells, et al. (2000a), despite their study considering multiple teeth: one sample by Lin, et al. (2010a) and three samples by Soori, et al. (2020). A total of 10 slices were used from two teeth to assess the emissivity of internal enamel and dentin from healthy and caries-affected teeth, and caution is needed when reviewing the outcomes from the slices as their independence will not be as great as that of 10 individual teeth. To the author’s knowledge, assessment of internal sound and carious mineralized tissue has not previously been reported from empirical data as undertaken in this study, although an assumed emissivity of 0.98 was used by Kaneko, et al. (1999) when assessing the feasibility of caries-detection using thermal data. The overall mean emissivity values of healthy enamel were found to be similar, at 0.97 for the internal flat surface and 0.96 for the external curved surface, and had the greatest mean emissivity value of any of the mineralized tooth-tissue assessed. This is similar to previous reports ranging from 0.96 to 0.98 and higher than others with a range of 0.65–0.9 (Table 1). Sound crown dentin tended to have a higher mean emissivity value (0.94) than root dentin (0.93), but not enamel, and the root face had the lowest mean emissivity value (0.84) of all sound mineralized tooth tissue. Both caries-affected enamel (0.82) and dentin (0.73) had lower emissivity values than their sound equivalents.

This can be attributed to different tissue compositions and structures, with enamel having a higher mineral content (95%) than dentin (60%) and dentin being composed of mineralized tubules rather than prisms and inter-prismatic crystals, as seen in enamel. Enamel will have natural surface irregularities seen as perikymata as well as internal irregularities from the prisms and inter-prismatic crystals. These surface textures appear to emit infrared radiation in a similar way, despite one being an internal surface and one an external surface. Assessment of an area of tissue may also account for this as a local change may be compensated for within the area calculation, compared to a spot-measurement as undertaken by Kells, et al. (2000a). The duration for which the teeth used in this study had been in the oral environment after eruption was unknown, and, thus, the degree of maturity of enamel was also unknown, which may affect the mineral content, as enamel increases in mineral composition following eruption.

The root-face returns the lowest value of all healthy mineralized tooth-tissue assessed and not only has a greater curvature than the flat surface of the cut crown-dentin and root-dentin but may also contain remnants of the cementum which has a reduced mineral content at 50% compared to the rest of dentin. There may be soft-tissue traces from the periodontal ligament as well, which influences the activity of the radiant energy.

The crown-dentin and root-dentin values are slightly higher than the published values of 0.8, produced from comparison with a black paint assumed to have an emissivity of 1 (Neev, et al., 1993). This is very improbable, as a perfect blackbody is empirically unlikely. The temperature of the assessment was also unknown. All tissue samples are non-homogenous and will vary in mineral composition and structure, all of which can affect the emissivity values and may account for some of the variations and demonstrates the need to assess each tooth.

Once dental caries demineralizes the tooth tissue, the mineral content changes and emissivity assessment may offer a diagnostic option early in the disease process. The lesion assessed in this study was advanced. However, attempts to assess caries due to thermal changes have been challenging, and the assessment of the emissivity may be sufficient to demonstrate early changes in tissue composition. The caries tissues all produced lower emissivity values than healthy tissues in thermal equilibrium. Occlusal caries assessment may present challenges from increased curvature of the cusps and the fissures which trap the radiation, and further studies are needed to explore emissivity further as a diagnostic aid.

### 4.1 Acceptance of methodology

The Tape method of assessing emissivity was simple and cheap and would be recommended for providing actual emissivity values for tooth tissue in any *in vitro* study, allowing consistency of methodology between research groups to report absolute temperatures. The emissivity of this Tape has been validated by the British Standards, and no independent evaluation of emissivity was carried out. In this study, the Tape was placed on the whole tooth sample and by the side of the slices. This may be criticized as the Tape was not physically on the slices. However, the slices were 1 mm thick and reached thermal equilibrium within 6 min. It is acknowledged that two teeth have been prepared to produce 10 slices for the assessment of internal emissivity of enamel and dentin and one tooth for the assessment of caries, which reduces the independence of outcomes described.

There were no measurements of the mineral density of any tissue, and, as such, the stage of demineralization is unknown for enamel and dentin and can be explored in a further study, having established the principle.

## 5 Conclusion

The method of calculation was cheap, simple and practical and can improve emissivity acquisition for comparison of absolute temperatures between studies evaluating thermal safety concerns for dental procedures and may offer a diagnostic aid in detecting demineralization and caries of tooth tissue.

Enamel had a high emissivity, which was similar whether from the internal flat surface of sliced enamel or the external curved enamel surface of a whole tooth and was reduced in caries-affected enamel.

Dentin also had a high emissivity (but not as high as enamel), which varied with location, with crown dentin being the highest compared to root dentin, and the root face had the lowest emissivity value but was still a good emitter of infrared radiation. Dentin emissivity was similarly reduced when affected by caries.

## Data Availability

The raw data supporting the conclusion of this article will be made available by the authors, without undue reservation.
